# Effect of Silver Nano-particles on Tensile Strength of Acrylic Resins

**DOI:** 10.15171/joddd.2015.008

**Published:** 2015-03-04

**Authors:** Tahereh Ghaffari, Fahimeh Hamedi-rad

**Affiliations:** ^1^Dental and Periodontal Research Center, Tabriz University of Medical Sciences, Tabriz, Iran; ^2^Assistant Professor, Department of Prosthodontics, Faculty of Dentistry, Tabriz University of Medical Sciences, Tabriz, Iran

**Keywords:** Acrylic resin, nano-particle, silver, tensile strength

## Abstract

***Background and aims.*** Polymethyl methacrylate (PMMA) is widely used for the fabrication of removable prostheses. Silver nano-particles (AgNps) have been added to PMMA because of their antimicrobial properties, but their effect on the mechanical properties of PMMA is unknown. The aim of this study was to investigate the effects of AgNps on the tensile strength of PMMA.

***Materials and methods.*** For this study, 12 specimens were prepared and divided into two groups. Group 1 included PMMA without AgNps and group 2 included PMMA mixed with 5 wt% of AgNps. Tensile strength of the specimens was measured by Zwick Z100 apparatus. Statistical analysis was carried out by SPSS using t-test. Statistical significance was defined at P<0.05.

***Results.*** This study showed that the mean tensile strength of PMMA in group 2 was significantly lower than that in group 1. Therefore, the tensile strength decreased significantly after incorporation of silver nano-particles.

Conclusion. Within the limitations of this study, tensile strength of acrylic resin specimens was influenced by silver nano-particles.

## Introduction

Polymethyl methacrylate (PMMA) is one of the most widely used materials in prosthodontics because of favorable esthetics and desirable characteristics, such as easy handling.^1^ Acrylic resins have excellent esthetic properties, adequate strength, low water sorption, low solubility, and lack of toxicity. They can reproduce surface details accurately and can be easily repaired. Although the properties of these materials are not ideal altogether, the desirable features mentioned above account for their popularity.^2-5^ However, it has some limitations. With use of PMMA as a denture base material, disadvantages such as relatively poor mechanical properties have been reported.^6-8^ However, some important disadvantages of PMMA, such as high coefficient of thermal expansion and relatively low modulus of elasticity, are intrinsic properties of the material.^3^ Mucosal irritation caused by the release of PMMA has also been reported.^1^

Generally, several techniques have been investigated to improve the impact properties of PMMA for development of an alternative material to PMMA, chemical modification of PMMA such as creating transverse bands, or the addition of a rubber graft copolymer, and the reinforcement of PMMA with other materials such as carbon fibers, glass fibers and ultra high-modulus polyethylene, tin or aluminum.^3,9^

The silver nano-particles (AgNps) are one of the most commonly used nano-particles because of their ductility, electrical and thermal conductivity, and antimicrobial activity.^10-13^ They have shown antimicrobial effects on many microorganisms, such as* E. coli, Staphylococcus aureus, Staphylococcus epidermidis, Candida albicans and Streptococcus mutans.*^14-17^ Therefore, it seems that use of AgNps in acrylic resins induces antimicrobial properties in these materials.^18^ In addition, release of silver from polymers containing silver nano-particles is more effective than polymers using silver in micrometer dimensions.^19^ Since nano-particles have the potential to impart ‘mechanical properties’ to some dental materials,^20^ one might assume that addition of AgNps to acrylic resins affects their mechanical characteristics. Therefore, although addition of AgNps has antimicrobial advantages, we should also be concerned about its effects on the tensile strength of PMMA.

Although previous studies have been conducted on metal fillers including tin, Al_2_O_3_, copper, and silver with weight percentages of 5%, 15%, and 20%,^6,7,13^ we used 5 wt% of nanosilver in order to minimize the probable unfavorable changes in mechanical and chemical properties of the denture acrylic base.

The aim of this study was to investigate the effect of AgNps on the tensile strength of one type of self-cured polymethyl methacrylate.

The null hypothesis was that addition of 5 wt% of AgNps to PMMA does not affect the tensile strength of PMMA.

## Materials and Methods

At first, the proportional amounts of acrylic resin powder and nano-silver (model no: SPA00601, Iran) were weighed by an accurate digital weighing machine and mixed in an amalgamator for achieving a homogeneous mixture.

The method used in this study followed ISO527:2000^21^ standards for denture base resins. Dimension of the specimens was 200×20(±0.2) ×2(±0.2) mm for tensile strength. One commonly used brand of heat-cured acrylic resin, Triplex (Ivoclar Vivadent AG, Lichtenstein), was used in this study. The number of specimens in each group was determined through a pilot study. After probing the pilot study, one test was carried out, which consisted of the following two groups, each with 6 specimens.

1. Triplex containing 0.00% of AgNps (control group)
2. Triplex containing 5% of AgNps

The specimens were prepared according to the order of manufacture, through denture baking with flasking, and processed well by very fine particles of Emery paper. The size of all the specimens was measured by a micrometer accurate to ±0.01 mm.

**Figure 1.**
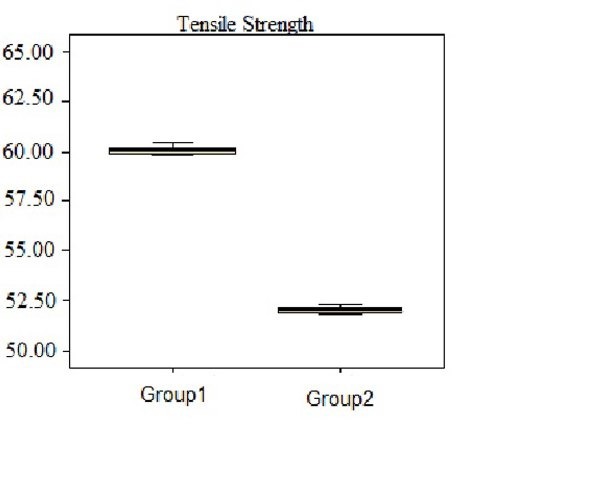
“Universal tensile strength measurement” apparatus with a cubic specimen.

The rectangular cubic specimens were placed in relevant points on a universal tensile strength measurement apparatus (Zwick Z100, Germany) (Figure 1) and fixed by levers. The apparatus exerted a tensile force (N) at a strain rate of 1 mm/min on each specimen until the specimen fractured. Force at the time of fracture was recorded. Considering the cross-sectional area of each sample, the tensile bond strength based on MPa was calculated using the following formula:

Bond strength (MPa) =Amount of force (N)/Cross-section surface area (mm^2^)

After examining the samples in group 1, the samples in group 2 were studied, and their tensile strength was also recorded. Following data collection, statistical analysis was carried out by SPSS (SPSS Inc., Chicago, IL, USA), using t-test. Statistical significance was defined at P<0.05.

## Results

**Figure 2.**
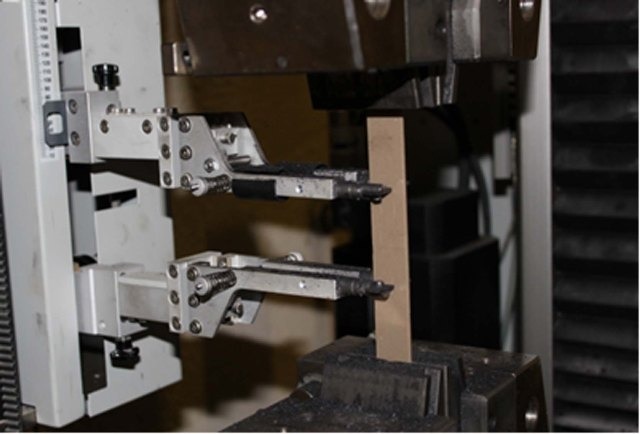
Mean value, domain, and standard deviation of tensile strength (MPa) in groups 1 and 2.

For each group, the standard deviations and the means were calculated for the tensile strength. The maximum tensile strength of the two groups was found in group 1. Addition of 5% AgNps to Triplex significantly decreased the tensile strength (Figure 2 and Table 1). The t-test analysis showed that this difference was statistically significant (P=0.000).

**Table 1:** Mean value, standard deviation, minimum, and maximum of tensile strength (MPa) in groups 1 and 2

					95%Confidence Interval for Mean			
	N	Mean	SD	Std. Error	Lower Bound	Upper Bound	Min	Max
Group 1	6	60.1000	.25298	.10328	59.8345	59.80	59.80	60.50
Group 2	6	52.0333	.17512	.07149	51.8496	51.80	51.80	52.30
Total	12	56.0667	4.21778	1.21778	53.3868	51.80	51.80	60.50

## Discussion

The results of this study indicated that incorporation of AgNps significantly decreased the tensile strength of PMMA, which might be attributed to less nano-silver particles per unit area of the PMMA matrix because of larger nano-silver particle size. This may also enhance the chances of void formation from entrapped air and moisture and incomplete wetting of the nanoparticles by resin. Therefore the net effect of embedding metal nanoparticles was to weaken the polymer. Similar reasons were cited by Sehjpal and Sod,^22^ who reported that addition of silver, aluminum or copper powder to PMMA at a concentration of 25% by volume significantly decreased (by as much as 35%) the tensile strength of the acrylic resin polymer.

Incorporation of NP (nano-particles) causes these particles to agglomerate and aggregate. The agglomerated compounds can act as stress-concentrating centers in the matrix and adversely affect mechanical properties of the polymerized material.^23^ On the other hand, one of the problematic issues in incorporating NP into acrylic resin is the lack of chemical bond between inorganic materials such as AgNps and PMMA. To improve bonding between metal and resin some chemicals such as 4-methacryloxyethyl trimellitate anhydride (4-META) and g-methacryloxypropyltrimethoxysilane (g-MPS) have been used.^24,25^ Accordingly, we can extrapolate that by finding out more appropriate substances as coupling agents between AgNps and PMMA, it might be possible to decrease its deleterious effects on mechanical properties.

However, it is obvious that the degree of nano-particles’ dispersion in the PMMA matrix is affected by the chemical composition of this acrylic resin. Probably, at high concentrations, particle dispersion and chemical interactions between PMMA and AgNps are low, resulting in a decrease in tensile strength.

Consequently, in a study by Sodagar et al use of 0.05% concentration of AgNps in Rapid Repair decreased flexural strength significantly. Nevertheless, for 0.2% concentration of AgNps in Rapid Repair, as well as 0.2% and 0.05% in Selecta Plus, the effect on flexural strength was insignificant.^26^ Therefore, the type of acrylic resin is an important factor in the effect of AgNps on flexural strength of PMMA.

In a study by Kassaee et al, 0.5% concentration of AgNps was used, which resulted in no significant effect on the flexural strength of PMMA.^16^This concentration is lower than that employed in the present study (5%).

The results of this study are not consistent with those of She, in which addition of AgNps to denture base inhibited the growth of* Streptococcus mutans* and* Candida albicans *but did not have any significant effect on the mechanical properties of the denture base resin.^15^

It is noteworthy that the content of nano additives is of critical importance. Even in modified polymeric materials to which TiO_2_ NP addition reinforced the matrix such as silicon elastomer or nano composites, when NP exceeded a particular percentage of 2.5 wt% as for example in elastomer matrix or 0.4 wt% in a nanocomposite, an opposite trend was observed and the value of tensile strength decreased in the elastomeric material and solubility increased in the dental composite.^23^

Since this research was limited to one type of acrylics and one concentration of AgNps, further studies are necessary to evaluate the effects of different concentrations of AgNps on other types of acrylics. It should be kept in mind that AgNps might affect tensile strength of some types of acrylics and therefore their advantage of antimicrobial properties should be weighed up against probable influence on tensile strength or some other physical and mechanical properties. Therefore, it seems that addition of AgNps to PMMA for antimicrobial activity would be beneficial but it should be performed with care and after finding special concentration of it with no affect on the other physical and mechanical properties of the acrylic.

## Conclusion

Since adding nano-silver particles by 5 wt% decreased the tensile strength of the acrylic resin, it is recommended that this study be repeated with different mixtures of various percentages of nano-silver particles in order to determine the best weight percentage that minimizes this disadvantage.
